# Isolation and Functional Determination of SKOR Potassium Channel in Purple Osier Willow, *Salix purpurea*

**DOI:** 10.1155/2021/6669509

**Published:** 2021-02-25

**Authors:** Yahui Chen, Xuefeng Peng, Jijie Cui, Hongxia Zhang, Jiang Jiang, Zhizhong Song

**Affiliations:** ^1^Collaborative Innovation Center of Sustainable Forestry in Southern China of Jiangsu Province, Nanjing Forestry University, Nanjing, Jiangsu, China; ^2^The Engineering Research Institute of Agriculture and Forestry, Ludong University, Yantai, Shandong, China; ^3^Molecular Testing Laboratory of New Plant Varieties in Southern China of State Forestry Administration, Nanjing, Jiangsu, China; ^4^Key Laboratory of Molecular Module-Based Breeding of High Yield and Abiotic Resistant Plants in Universities of Shandong (Ludong University), Yantai, Shandong, China; ^5^Department of Plant Science, University of Cambridge, Cambridge CB2 3EA, UK

## Abstract

Potassium (K^+^) plays key roles in plant growth and development. However, molecular mechanism studies of K^+^ nutrition in forest plants are largely rare. In plants, *SKOR* gene encodes for the outward rectifying Shaker-type K^+^ channel that is responsible for the long-distance transportation of K^+^ through xylem in roots. In this study, we determined a Shaker-type K^+^ channel gene in purple osier (*Salix purpurea*), designated as *SpuSKOR*, and determined its function using a patch clamp electrophysiological system. SpuSKOR was closely clustered with poplar PtrSKOR in the phylogenetic tree. Quantitative real-time PCR (qRT-PCR) analyses demonstrated that *SpuSKOR* was predominantly expressed in roots, and expression decreased under K^+^ depletion conditions. Patch clamp analysis via HEK293-T cells demonstrated that the activity of the SpuSKOR channel was activated when the cell membrane voltage reached at -10 mV, and the channel activity was enhanced along with the increase of membrane voltage. Outward currents were recorded and induced in response to the decrease of external K^+^ concentration. Our results indicate that SpuSKOR is a typical voltage dependent outwardly rectifying K^+^ channel in purple osier. This study provides theoretical basis for revealing the mechanism of K^+^ transport and distribution in woody plants.

## 1. Introduction

Plants need to absorb the required K^+^ from the soil through plant roots and, then, distribute them to different organs to meet the normal growth and development [1–6]. Previous studies demonstrated that there are two kinds of mechanisms of K^+^ uptake in plants. Mechanism I is the high-affinity K^+^ absorption system, which plays a major role when the external K^+^ concentration is less than 200 *μ*mol·L^−1^. Mechanism II is the low affinity K^+^ absorption system, which plays key role when the external K^+^ concentration is more than 1 mmol·L^−1^ [1, 7, 8]. The long-distance distribution and dynamic balance of·K^+^ in plants are mainly mediated by various K^+^ channels located in the plasma membrane. According to the sequence, structure, and function of these channel proteins, they can be divided into three categories: Shaker family channels, TPK family channels, and other potassium channels [2, 4, 6, 9]. Among them, Shaker-type K^+^ channels are the most thoroughly studied. The affinity constant of Shaker-type K^+^ channels to substrate K^+^ is about tens of millimoles, which is a typical low affinity and high-throughput K^+^ channel, and plays an important role in plant K^+^ nutrition efficiency [2, 3, 5, 6].

Since Anderson et al. [10] and Sentenac et al. [11] reported the Shaker-type K^+^ channels KAT1 and AKT1 in *Arabidopsis thaliana*, more than 40 Shaker-type K^+^ channels have been found in different plants during the recent 30 years [2, 3, 12–16]. According to the voltage dependence and the different movement direction of K^+^ in the transmembrane, Shaker-type K^+^ channels can be divided into three types: inward rectifier, outward rectifier, and weak rectifier (bidirectional rectifier). In *Arabidopsis*, the inward rectifying K^+^ channels include AKT1, SPIK, KAT1, and KAT2 [10, 11, 17, 18]. SKOR and GORK are typical outward rectifier K^+^ channels [9, 14], and AKT2/3 is a typical weak rectifier K^+^ channel [19, 20].

In particular, *SKOR* gene encodes a class of outward rectifying Shaker-type K^+^ channel. In *Arabidopsis*, the molecular mechanism of the SKOR channel physiological function is more detailed: *AtSKOR* is mainly located in the columella and pericycle parenchyma cells of the root, which is responsible for the K^+^ release into the xylem sap, thus realizing the long-distance transportation of K^+^ ions through the xylem. Meanwhile, it was found that the lack of AtSKOR channel function could reduce the K^+^ content in the shoot by about 50% and the plant growth and development [9]. In recent years, SKOR has been widely studied in rice [14], muskmelon [15], *Puccinellia tenuiflora* [21], and *Zygophyllum Xanthoxylum* [22]. However, the function of SKOR channels in woody plants is largely rare.

As one of the most popular diploid willow plants, purple osier plays an important role in soil and water conservation, shelter forest, and biomass energy, and its genome has been successfully sequenced [23, 24]. The molecular basis and mechanisms of K^+^ nutrition and homeostasis in willow are still unknown. In this study, we isolated a Shaker-like K^+^ channel gene, *SpuSKOR*, from purple osier, and analyzed their function via patch clamp electrophysiological system, which provided a theoretical basis for revealing the mechanism of K^+^ transport and distribution in woody plants.

## 2. Material and Methods

### 2.1. Plant Material and Growth Condition

Purple osier plants used throughout this study were collected as previously described in Liang et al. [25]. 1-year-old female purple osier cutting asexual cloning plants were grown in advance in 1/2MS liquid medium (the control, [26]) in a growth chamber with 12 h light at 25°C followed by 12 h dark at 20°C (with 60% relative humidity) for 2 weeks and, then, transferred into the plastic containers that being suffered to different treatments, based on 1/2MS liquid medium. Leaf, stem, root, full blooming flower, and mature fruit tissues were collected from the same plant and frozen immediately in liquid nitrogen for further quantitative real-time PCR (qRT-PCR) analyses.

K^+^ deficiency treatment was carried out as previously described in Liang et al. [25]. 1-year-old female purple osier cutting asexual cloning plants were subjected to K^+^ deficiency and collected at 0 h, 4 h, 12 h, 36 h, and 72 h before qRT-PCR analysis.

### 2.2. Identification of SpuSKOR Gene

Genome information of purple osier willow was screened from The Plant Genomics Resource of Phytozome 12 (https://phytozome.jgi.doe.gov/pz/portal.html). BLAST searches against the genome database were carried out with the full-length of *Arabidopsis* SKOR (AT3G02850) protein sequence as reference, to obtain the *SKOR* gene from purple osier. The amino acid sequence of the candidate purple osier SKOR protein was verified using the Pfam (http://pfam.xfam.org) and InterProScan 4.8 (http://www.ebi.ac.uk/Tools/pfa/iprscan/), to confirm the existence of typical K^+^ channel domains. Gene ID, location, coding sequence, amino acid sequence, and intron numbers were gathered on Phytozome Genomics Resources.

### 2.3. Phylogenetic Analysis of Known Plant SKOR Proteins

The full-length SKOR protein sequences of purple osier, *Arabidopsis*, rice, maize, soybean, tomato, grape, peach, apple, and poplar were downloaded from the Phytozome Genomics Resources, respectively, and pear SKOR protein was downloaded from Pear Genome Project (http://peargenome.njau.edu.cn/). A neighbor-joining method phylogenetic tree was constructed as previously described in Tamura et al. [27, 28], via using the ClustalX2.1 and MEGA7.0 software.

### 2.4. RNA Extraction and Quantitative Real-Time PCR Assay

As previously described in Liang et al. [25], RNA extraction was carried out using MiniBEST Plant RNA Extraction Kit (TaKaRa, Dalian, China), and the 1st cDNA was synthesized using PrimeScriptTM RT reagent Kit (TaKaRa, Dalian, China). Specific primers for SpuSKOR and Ubiquitin control gene were designed from NCBI/Primer-BLAST online server (Table 1). qRT-PCR was performed on 7500 Real-Time PCR System (Applied Biosystems, New York, USA) as described by Liang et al. [25] and You et al. [28]. Using SYBR Premix Ex Taq reaction kit (TaKaRa, Dalian, China), qRT-PCR efficiency and the starting template concentration were calculated according to the description of Song et al. [28–30]. The relative expression levels of *SpuSKOR* were presented after normalization to the internal control *SpuUbiquitin* from three independent biological repeats [25].

### 2.5. Patch Clamping Analysis

The recombinant plasmid pTracer-CMV3-*SKOR* was constructed by cloning the CDS region of *SpuSKOR* gene into pTracer-CMV3 vector [12], using the forward primer (Table 1, *Kpn* I site was introduced and underlined) and reverse primer (Table 1, *Not* I site was introduced and underlined). The electrophysiological function of *SpuSKOR* was carried out by patch clamp system as described by Su et al. [12]. The purified and concentrated pTracer-CMV3-*SKOR* plasmid was transfected into HEK293-T cells (ATCC company, USA). The cells labeled with green fluorescence were selected and being detected by PCLAMP 10.0 device (Axon, USA). The current signal of SpuSKOR channel was collected by PCLAMP 10.0 and then being analyzed via the Sigmaplot 10.0 software. The external K^+^ concentration in the extracellular fluid was set of 0, 10, 50, and 100 mmol·L^−1^, each concentration with 6 cells.

### 2.6. Statistical Analysis

All data were statistically analyzed using independent samples *t*-test in the SPSS 13.0 software (SPSS Chicago, Ilinois, USA). *Asterisks* indicate statistical differences between plants under control and stress treatment (^∗^*P* < 0.05, ^∗∗^*P* < 0.01).

## 3. Results

### 3.1. Identification of SpuSKOR in Purple Osier Willow

**Figure 1:**
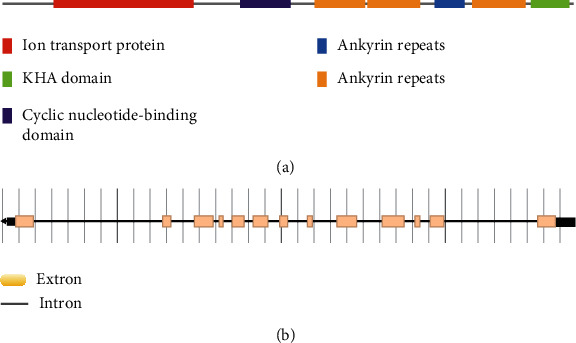
Domain prediction and gene structure analysis. (a) Domain prediction of SpuSKOR protein. (b) Gene structure analysis of *SpuSKOR* gene.

By BLAST searching of the Phytozome Genomics Resources of Purple Osier Willow, a putative *SKOR* channel encoding gene was identified, entitled as *SpuSKOR*, which contains a coding sequence of 3099 bp in nucleotides that encodes 1032 amino acids. Protein domain verification analyses showed that all of them contain six ion transmembrane domains (PF00520), three ankyrin domain (PF12796), one cyclic nucleotide-binding domain (PF00027), and one KHA dimerisation domain (PF11834), which belongs to the classic plant potassium channels (Figure 1(a)). Gene structure analysis showed that *SpuSKOR* had 16 introns that varied distinctly in length (Figure 1(b)).

### 3.2. Phylogenetic and Protein Motif Analysis of SKOR Proteins

**Figure 2:**
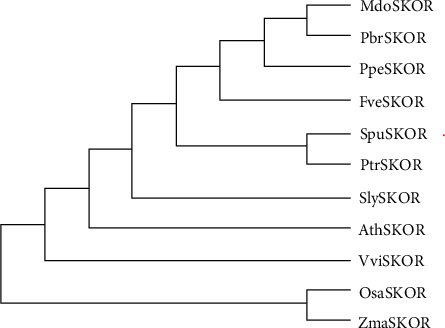
Phylogenetic tree of SKOR proteins from different plants. A maximum likelihood (ML) tree was constructed by multiple alignment of SKOR proteins in purple osier, *Arabidopsis*, rice, maize, soybean, tomato, grape, peach, apple, and poplar using the ClustalX2.1 and MEGA7.0 software. Information of SKOR proteins from sequenced plant was listed in Supplemental Table 1. The SpuSKOR protein was labeled with red dot.

To confirm the evolutionary relationship of SKOR proteins of 11 sequenced plants, a maximum likelihood (ML) phylogenetic tree was generated. The amino acid sequences of 11 SKOR proteins share an overall identity of 53.39% (data not shown). Notably, purple osier willow and poplar belong to the same family of Salicaceae; SpuSKOR was closely clustered with PtrSKOR from poplar in the phylogenetic tree (Figure 2). Rice and maize belong to the same family of Gramineae; OsaSKOR and ZmaSKOR were closely clustered together. Moreover, all Roseaceae orthologs from apple (MdoSKOR), pear (PbrSKOR), peach (PpeSKOR), and strawberry (FveSKOR) have the closest genetic relationships (Figure 2).

### 3.3. Expression Profiles of SpuSKOR

**Figure 3:**
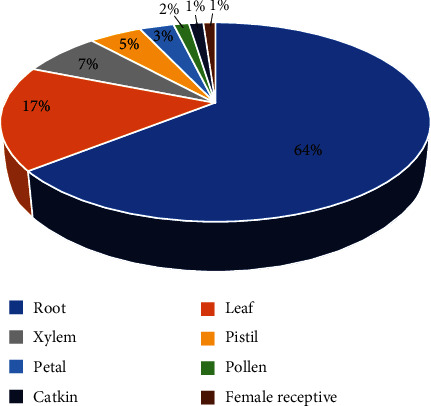
In silico transcriptomic expression pattern of *SpuSKOR* in different tissues/organs of purple osier. The expression levels (RPKM) of *SpuSKOR* were directly downloaded from Phytozome Genomic Resources (purple osier).

**Figure 4:**
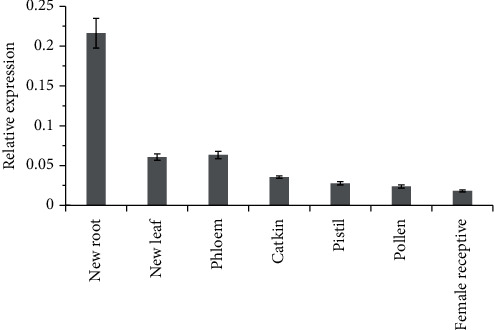
qRT-PCR analysis of *SpuSKOR* in different tissues/organs of purple osier. 1-year-old female purple osier plants were grown in 1/2MS liquid medium in a growth chamber with 12 h light at 25°C followed by 12 h dark at 20°C (with 60% relative humidity). Different tissues/organs were collected from the same plant on April 20, 2019, and frozen immediately in liquid nitrogen for RNA extraction and quantitative real-time PCR. Data are the means of values obtained from three independent replicates ± SE.

The transcriptomic data of purple osier was obtained from Phytozome online database. In general, the percentages of SpuSKOR expression in different tissues and organs are as follows: the maximum expression was detected in roots (64%), followed by leaf (17%), xylem (7%), pistil (5%), petal (3%), pollen (2%), catkin (1%), and female receptive (1%) (Figure 3). qRT-PCR was further performed to determine the expression profiles of *SpuSKOR* in different tissues of 1-year-old female purple osier. Results showed that *SpuSKOR* was unevenly expressed in the tested organs, and mainly expressed in roots, followed by leaves, phloem, catkin, pistil, pollen, and female receptive (Figure 4), which was consistent with the transcriptomic expression status.

**Figure 5:**
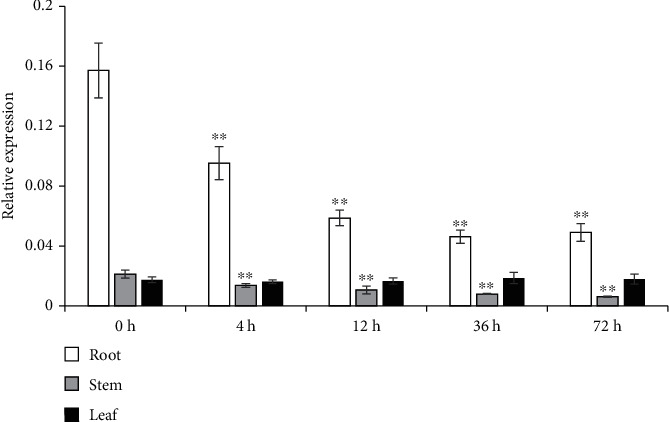
Expression changes of *SpuSKOR* under K^+^ deficiency. 1-year-old purple osier plants were exposed to K^+^ deficiency for 4 h, 12 h, 36 h, and 72 h, respectively, before examination. The relative expression level of *SpuSKOR* was presented after normalization to the internal control. Data are the means of values obtained from three independent replicates ± SE.

To investigate the role of *SpuSKOR* in maintaining K^+^ homeostasis in purple osier, we analyzed the expression profiles of *SpuSKOR* in roots, leaves, and stems of 1-year-old purple osier seedlings under K^+^ depletion. Results showed that expression of *SpuSKOR* in roots was consistently decreased until 12 h and, then, kept the same. Although expression level was largely low in stems and leaves, *SpuSKOR* in stems was steadily reduced until 72 h, while *SpuSKOR* in leaves was not affected under K^+^ deficiency (Figure 5).

### 3.4. Patch Clamp Determination of SpuSKOR in HEK293-T Cells

**Figure 6:**
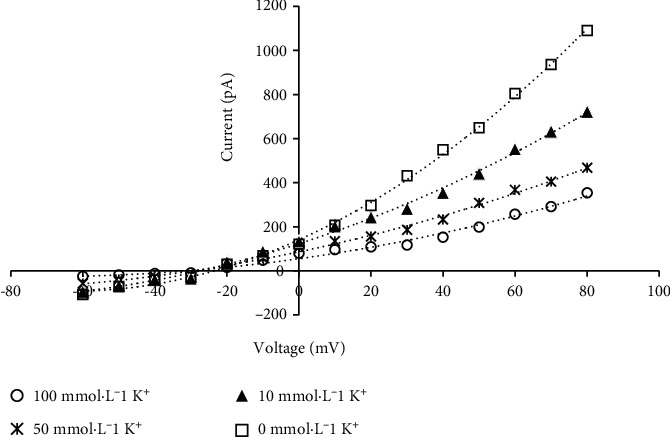
Curves of the current-voltage relation in response to different extracellular K^+^ concentrations via patch clamp system. Green fluorescence-labeled HEK293-T cells were selected and being detected by PCLAMP 10.0 device. The current signal of the SpuSKOR channel was collected by PCLAMP 10.0 and then being analyzed via the Sigmaplot 10.0 software. The external K^+^ concentration in the extracellular fluid was set of 0, 10, 50, and 100 mmol·L^−1^. Data are shown as the means of values obtained from 6 independent cells.

Taking HEK293-T cells transfected with pTracer-CMV3 empty vector were used as the control, pCLAMP 10.0 patch clamp system was used to record the characteristic curves of current and membrane voltage of pTracer-CMV3-*SKOR* under different extracellular K^+^ concentrations. Results showed that cells expressing pTracer-CMV3-*SKOR* exhibited outward rectifying currents, without deducting the control background currents, and the outward current increased with the decrease of extracellular K^+^ concentration (Figure 6). Notably, the lowest current was recorded when the extracellular K^+^ concentration was set at 100 mmol·L^−1^, while the highest current was recorded when the extracellular K^+^ concentration was set at 0 mmol·L^−1^ (Figure 6).

In addition, when the cell membrane voltage was at -10 mV, the SpuSKOR channel was activated and the outward rectifying current appeared. The more positive the voltage was, the stronger the outward rectifier current was (Figure 6).

## 4. Discussion

As one of the most important cation elements in plant cells, K^+^ is closely related to plant growth and development [1–4, 6]. K^+^ fertilizer favorably contributes to plant growth, flowering, wood quality, and yield [2–6]. Molecular mechanisms towards K^+^ nutrition in perennial forest plants are largely rare.

In plants, *SKOR* genes encode a class of outward rectifying Shaker-like K^+^ channel proteins, which play an important role in plant growth and development [4, 9, 30]. To date, there are more than 650 species of *Salicaceae* in the world. However, no SKOR channel protein in *Salicaceae* has been reported. Although the core transmembrane regions of SKOR homologous proteins in woody plants of different families and genera have very high sequence consistency (data not shown), there are differences in the genetic evolution relationship (Figure 2). SKOR homologous proteins of the same family and genus have relatively high consistency and are more similar in genetic evolution distance (Figure 2). In particular, SpuSKOR and poplar PtrSKOR are closely clustered in the phylogenetic tree, and they have high amino acid sequence identity of 94.54% (Supplemental Figure 1). The highly similar amino acid sequence and protein structure (Supplemental Figure 2) indicating that they might have similar physiological functions. Analyzing the function of SpuSKOR channel in purple osier provides theoretical support for studying the function of SKOR homologous proteins in *Salicaceae*.

The cell-specific expression patterns of these *SKOR* genes may be essential for the specific functions of the channels and plant growth. In *Arabidopsis* and rice, *SKOR* is specifically expressed in roots [9, 14, 19], while in muskmelon and *Zygophyllum xanthoxylum*, *SKOR* is ubiquitously expressed and the maximum expression was observed in roots [15, 22]. In this study, *SpuSKOR* was mainly expressed in the roots but slightly expressed in the other parts of purple osier (Figure 4), which was consistent with previous reports in other plants [15, 21, 22], but was slightly differed from *Arabidopsis* and rice [9, 14]. Nonetheless, our findings confirmed again that *SKOR* genes mainly play indispensable roles in K^+^ nutrition and ion dynamics in plant roots.

Favorably, electrophysiological studies may explain the functional characteristics and regulatory mechanisms of plant K^+^ channels measured in vitro. In particular, patch clamp and two electrode voltage clamp are the two most effective systems to record the characteristic curves of current, under distinct membrane voltage, and to determine the physiological function of ion channels [2, 3, 9, 14, 15, 17, 18, 19, 21, 22]. In terms of physiological function, plant SKOR proteins belong to a typical outward rectifying Shaker-type K^+^ channel with K^+^ selectivity. It has been confirmed in *Arabidopsis*[9, 19] and muskmelon [15] by using *Xenopus* oocytes and double electrode voltage clamp technology, patch clamping studies of SKOR channels are rare. In the model organism *Arabidopsis*, Shaker-like outwards rectifying channel *AtSKOR* is expressed in the pericycle and the xylem parenchyma in roots. The activity of the AtSKOR channel is modulated by membrane potential, along with external K^+^ concentration. The AtSKOR channel opens at depolarized membrane potential [9]. In this study, patch clamp determination revealed that SpuSKOR had the characteristics of outward rectifying channel: K^+^ efflux current and voltage-dependent gated channel activity, and the channel activity is regulated by extracellular K^+^ concentration (Figure 6), which is similar to the K^+^ current characteristics of *Arabidopsis* AtSKOR and muskmelon CmSKOR [9, 13, 19]. However, there were significant differences in current intensity, current, and membrane potential curves, suggesting that the functions of woody plant SKOR channels are quite different from those of annual plants. Notably, SpUSKOR current is recorded at negative membrane voltage (-10 mV) (Figure 6); this was because these recorded currents are nonnormalized currents that without deducting the control background currents. Although the characteristics and regulation mechanism of SpuSKOR channel for K^+^ transport have not been carried out, this work provides theoretical basis and technical support for the study of the function of SKOR homologous proteins in woody plants.

Shaker-type channels play an important role in K^+^ homeostasis, osmotic regulation, and proton regulation and are regulated by abiotic stresses, including external K^+^ supply levels [5, 9, 14, 15, 19, 21, 22]. Purple osier plants have strong adaptability and play important roles in water and soil conservation, bioenergy, and shelter forest system construction [23, 24]. In this study, K^+^ deficiency significantly reduced the expression level of *SpuSKOR* (Figure 5), which was consistent with the previous studies in *Arabidopsis* [9], *Zygophyllum Xanthoxylum* [22], and muskmelon [15]. Therefore, we speculate that there is not sufficient K^+^ that could be transported to the upper part of the plant roots, under the condition of K^+^ deficiency, which reduces the demand of *SKOR* genes and causes the decrease of *SKOR* expression levels. Nonetheless, SpuSKOR is a typical voltage-dependent outward rectifying K^+^ channel in purple osier plants.

## 5. Conclusions

A Shaker-type K^+^ channel gene, *SpuSKOR*, was isolated and determined in purple osier. *SpuSKOR* was mainly expressed in roots and was downregulated by K^+^ deficiency. SpuSKOR is a typical voltage-dependent outward rectifying K^+^ channel in purple osier.

## Figures and Tables

**Table 1:** Primer sequences used in this work.

Purpose	Primer (5′-3′)	Amplicon (bp)
Amplification of *SpuSKOR* CDS	F: ATGGACGGTCATGTCAGTCACA R: TCAAGATAACTGATGTGTTCGA	3099
Specific expression primers of *SpuSKOR*	F: GAATCAGACGGTGATGATGAGAA R: ACCGGAGGAGACATACCGA	207
Specific expression primers of *SpuUbiquitin*	F: TGGGTTTGCTGGAGATGAT R: CAGTAGGAGAACTGGGTGC	156
Construction of pTracer-CMV3-*SKOR* plasmid	F: GACGGTACCATGGACGGTCATGTCAGTCACA R: GCGGCGGCCGCTCAAGATAACTGATGTGTTCG	3099

## Data Availability

The data used to support the findings of this study are included within the article.

## Supplementary Materials

Supplementary Materials Supplemental Figure 1: amino acid alignment of SpuSKOR and PtrSKOR proteins. Supplemental Figure 2: tertiary structure prediction of SpuSKOR and PtrSKOR proteins. Supplemental Table 1: information of SKOR proteins from sequenced plants.
