# Supplementary material for: Isolation and Functional Determination of SKOR Potassium Channel in Purple Osier Willow, Salix purpurea
Source: Int J Genomics. 2021 Feb 25;2021:6669509. doi: 10.1155/2021/6669509 (PMC7932800; doi:10.1155/2021/6669509)
Supplement: Supplementary Materials — Supplemental Figure 1: amino acid alignment of SpuSKOR and PtrSKOR proteins. Supplemental Figure 2: tertiary structure prediction of SpuSKOR and PtrSKOR proteins. Supplemental Table 1: information of SKOR proteins from sequenced plants. [file 6669509.f1.zip › Supplemental Figure 1 (1).pdf]

```
SpuSKOR   MDGHVSHREKNKKESDGDDEKEEEYEVQDFKDRIKSSRGSRFNLIEKEFGLVNNTESSSMTSWRRKLSRESVINGLRYVSSGFVIHPDNRWYRAWTKFIL   100
PtrSKOR   MDGHGNHRERNKKE.DSDDDGEEEYEVEDLKDRIKSSRGSRFNLIEKEFGLVNNNGSSSMTSWRRKLSRESVINGIRYVSSGFVIHPDNRWYRAWTKFIL    99
Consensus mdgh  hre nkke d dd  eeeyev d kdrikssrgsrfnliekefglvnn  sssmtswrrklsresving ryvssgfvihpdnrwyrawtkfil

SpuSKOR   LWAVYSSFFTPLEFGFFRGLPENLFILDIVGQVAFLLDIIFQFFIAYRDSQTYRTVYKRSPIALRYLKSHFIIDLLACLPWDIIYKVCGHKEEVRYLLWI   200
PtrSKOR   LWAVYSSFFTPMEFGFFRGLPENLFILDIVGQVAFLLDIILQFFIAYRDSQTYRTVYKRTPIALRYLKSHFIIDLLACLPWDIIYKACGHREEVRYLLWI   199
Consensus lwavyssfftp efgffrglpenlfildivgqvaflldii qffiayrdsqtyrtvykr pialrylkshfiidllaclpwdiiyk cgh eevryllwi

SpuSKOR   SLSRVRKVTGFFQNMEKDIRINYLFTRIVKLIVVELYCTHTAACIFYYLASTLPSSQEGYTWIGSLKMGDYSYTSFREIDIWKRYITSLYFAIITMATVG   300
PtrSKOR   RLSRVRKVTDFFQKMEKDIRINYLFTRIVKLIVVELYCTHTAACIFYHLATTLPSSQEGYTWIGSLKMGDYSYTSFREIDIWKRYTTSLYFAVITMATVG   299
Consensus  lsrvrkvt ffq mekdirinylftrivklivvelycthtaacify la tlpssqegytwigslkmgdysytsfreidiwkry tslyfa itmatvg

SpuSKOR   YGDIHAVNLREMIFVMIYVSFDMILGAYLIGNMTALIVKGSKTERFRDKMTDLIKYMNRNRLGKEIRNQIKGHVRLQYESSYTEASALQDLPISIRAKVS   400
PtrSKOR   YGDIHAVNLREMIFVMIYVSFDMILGAYLIGNMTALIVKGSKTEKFRDKMTDLIKYMNRNRLGKDIRNQIKGHVRLQYESSYTEASALQDLPISIRAKVS   399
Consensus ygdihavnlremifvmiyvsfdmilgaylignmtalivkgskte frdkmtdlikymnrnrlgk irnqikghvrlqyessyteasalqdlpisirakvs

SpuSKOR   QTLYTEYIEKVPLLKGCSAEFINQIVIRLHEEFFLPGEVIMEQGNVVDQFYFVCHGVLEEVGIGQDGSEETVKLLPPNSSFGEISILCNIPQPYTVRVCE   500
PtrSKOR   QTLYTEYIEKVPLLKGCSAEFINQIVIRLHEEFFLPGEVIMEQGNVVDQLYFVCHGVLEEVGIGQDGSEETVKLLPPNSSFGEISILCNIPQPYTVRVCE   499
Consensus qtlyteyiekvpllkgcsaefinqivirlheefflpgevimeqgnvvdq yfvchgvleevgigqdgseetvkllppnssfgeisilcnipqpytvrvce

SpuSKOR   LCRLLRIDKQSFSNILEIYFYDGRKILDNLLEGKESNLRDKQLESDITFHIGKQEAELALSVNNAAYHGDLYQLKGFIRAGADPNKTDYDGRSPLHLAAS   600
PtrSKOR   LCRLLRIDKQSFSNILEIYFYDGRKILDNLLEGKESNLRDKQLESDITFHIGKQEAELALRVNSAAYHGDLYQLKGFIRAGADPNRTDYDGRSPLHLAAS   599
Consensus lcrllridkqsfsnileiyfydgrkildnllegkesnlrdkqlesditfhigkqeaelal vn aayhgdlyqlkgfiragadpn tdydgrsplhlaas

SpuSKOR   RGYEDITLFLIQEGVDINIKDKFGNTPLLEAIKNGHDRVESLLFKQGAILNIDDAGSVLCRAVARGDSDFLKRVLSNGIDPNSKDYDHRTPLHVAASEGL   700
PtrSKOR   RGYEDITLFLIQEGVDINIKDKFGNTPLLEAIKNGHDRVESLLFKQGAILNIDDAGSVLCRAVARGDSDFLKRILSNGIDPNSKDYDHRTPLHVAASEGL   699
Consensus rgyeditlfliqegvdinikdkfgntplleaiknghdrvesllfkqgailniddagsvlcravargdsdflkr lsngidpnskdydhrtplhvaasegl

SpuSKOR   YLMAKLLIEAGACVFSKDRWGNTPLDEGRMCGSKNLIKLLEEAKSSQKLDFHYSTHETREKVLPKKCTIFPFHPRGSEEQRRPGVVLWVPDTMEELVKEA   800
PtrSKOR   YLMAKLLIEAGASVFSKDRWGNTPLDEGRMCGNKKLIKLLEEAKSSQKLEFHYSTHETTEKVLPKKCTIFPFHPR.AEEQRRPGVVLWVPNTMEELVKAA   798
Consensus ylmakllieaga vfskdrwgntpldegrmcg k liklleeakssqkl fhysthet ekvlpkkctifpfhpr  eeqrrpgvvlwvp tmeelvk a

SpuSKOR   SEQLQFPDGFCILTEDAGKILDVKMIDEGQKLYLTSDRTHQL                                                           842
PtrSKOR   SEQLQFPDGSCILSEDAGKILDVNMIDGGQKLYLTSDQTHYL                                                           840
Consensus seqlqfpdg cil edagkildv mid gqklyltsd th l
```
