# Supplementary material for: Isolation and Functional Determination of SKOR Potassium Channel in Purple Osier Willow, Salix purpurea
Source: Int J Genomics. 2021 Feb 25;2021:6669509. doi: 10.1155/2021/6669509 (PMC7932800; doi:10.1155/2021/6669509)
Supplement: Supplementary Materials — Supplemental Figure 1: amino acid alignment of SpuSKOR and PtrSKOR proteins. Supplemental Figure 2: tertiary structure prediction of SpuSKOR and PtrSKOR proteins. Supplemental Table 1: information of SKOR proteins from sequenced plants. [file 6669509.f1.zip › Supplemental Table 1.docx]

Supplemental Table 1. Information of SKOR proteins from sequenced plants

| Species | Protein | Gene ID | CDS (bp) | Amino acid No. |
| --- | --- | --- | --- | --- |
| Purple oiser | SpuSKOR | SapurV1A.0223s0270 | 2532 | 843 |
| Grape | VviSKOR | GSVIVT01030667001 | 2385 | 794 |
| Arabidopsis | AthSKOR | AT3G02850 | 2487 | 828 |
| Rice | OsaSKOR | LOC_Os06g14030 | 2577 | 858 |
| Soybean | ZmaSKOR | GRMZM2G310569_T01 | 2640 | 879 |
| Tomato | SlySKOR | Solyc11g011500 | 2490 | 829 |
| Poplar | PtrSKOR | Potri.017G135400 | 2526 | 841 |
| Peach | PpeSKOR | Prupe.3G164900 | 2493 | 830 |
| Pear | PbrSKOR | Pbr022827 | 2521 | 839 |
| Strawberry | FveSKOR | mrna30492.1-v1.0-hybrid | 3795 | 1264 |
| Apple | MdoSKOR | MDP0000263295 | 2523 | 840 |
