# Supplementary figures and images for: Isolation and Functional Determination of SKOR Potassium Channel in Purple Osier Willow, Salix purpurea
Source: Int J Genomics. 2021 Feb 25;2021:6669509. doi: 10.1155/2021/6669509 (PMC7932800; doi:10.1155/2021/6669509)

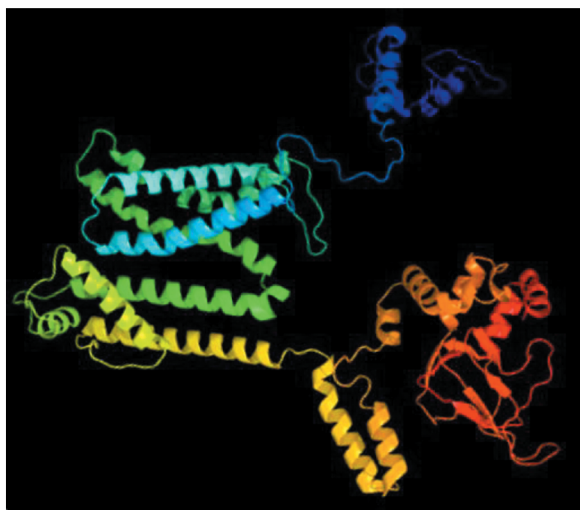

**SpuSKOR**

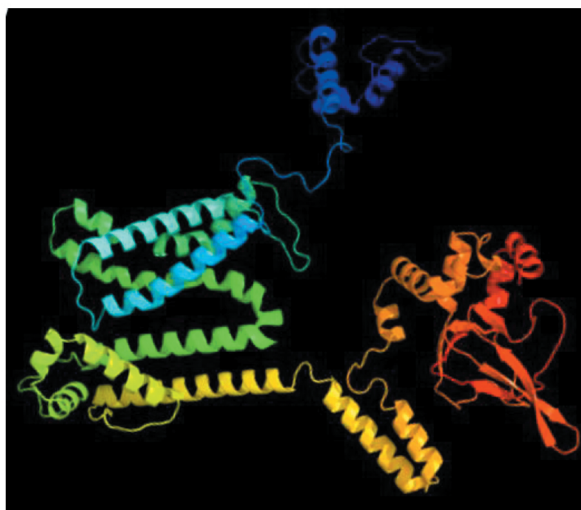

**PtrSKOR**

Supplement: Supplementary Materials — Supplemental Figure 1: amino acid alignment of SpuSKOR and PtrSKOR proteins. Supplemental Figure 2: tertiary structure prediction of SpuSKOR and PtrSKOR proteins. Supplemental Table 1: information of SKOR proteins from sequenced plants. [file 6669509.f1.zip › Supplemental Figure 2 (1).pdf]
